# Financial incentives and information provision for post-pandemic primary care quality recovery: a longitudinal study in Catalonia

**DOI:** 10.3399/BJGP.2025.0518

**Published:** 2026-04-21

**Authors:** Roger Esteban-Fabró, Ermengol Coma, Francesc X Marin-Gomez, Eduardo Hermosilla, Leonardo Méndez-Boo, Carolina Guiriguet, Gabriel Facchini, Concepción Violán, Catia Nicodemo, Josep Vidal-Alaball

**Affiliations:** 1 Fundació Institut Universitari per a la Recerca a l'Atenció Primària de Salut Jordi Gol i Gurina (IDIAP Jordi Gol), Barcelona, Spain; 2 Primary Care Services Information System (SISAP), Institut Català de la Salut (ICS), Barcelona, Spain; 3 SISAP Research Group (GR-SISAP), IDIAP Jordi Gol, Barcelona, Spain; 4 Intelligence for Primary Care Research Group, IDIAP Jordi Gol, Manresa, Spain; 5 Servei d’Atenció Primària Osona, Gerència Territorial de la Catalunya Central, ICS, Barcelona, Spain; 6 Equip d'Atenció Primària de Gòtic, ICS, Barcelona, Spain; 7 Department of Economics at Royal Holloway, University of London, London, UK; 8 Unitat de Suport a la Recerca Metropolitana Nord, IDIAP Jordi Gol, Mataró, Spain; 9 Nuffield Department of Primary Care Health Sciences, University of Oxford, Oxford, UK; 10 Business School, Brunel University of London, London, UK; 11 Unitat de Recerca i Innovació, Gerència d'Atenció Primària i a la Comunitat de la Catalunya Central, ICS, Manresa, Spain; 12 Department of Medicine, University of Vic-Central, University of Catalonia, Vic, Spain

**Keywords:** COVID-19, economic incentives, health policy, primary care, quality-of-care indicators

## Abstract

**Background:**

The COVID-19 pandemic disrupted primary care services worldwide, reducing quality of care. Understanding the impact of quality-improvement strategies can guide system resilience.

**Aim:**

To assess whether information provision and financial incentives to primary care professionals supported quality of care recovering to pre-pandemic levels after pandemic-related declines.

**Design and setting:**

This study involved descriptive analysis at the practice and aggregated indicator levels in Catalonia, Spain.

**Method:**

Data on 37 quality-of-care indicators from 287 primary care practices (5 250 531 adults) were analysed monthly from 2019 until 2024. Financial incentives were suspended in 2020; 13 indicators were reintegrated into pay-for-performance schemes in 2021 (early-incentivised indicators), while 24 remained out of the schemes until 2023 (late-incentivised indicators). Outcomes were annual changes in indicator results and variability, and time to recover pre-pandemic care-quality levels. A multivariate Cox model estimated the effect of incentives; *Κ*-means clustering identified practice recovery profiles.

**Results:**

In 2021, 11 (85%) of 13 of early-incentivised indicators started showing signs of recovery versus five (21%) of 24 late-incentivised indicators. By December 2024, recovery of pre-pandemic results occurred in 85% and 50% of early- and late-incentivised indicators, respectively, despite larger pandemic drops in early-incentivised indicators. Early incentivisation doubled the likelihood of recovery before 2023 (2.06 times), with effects varying by indicator type. However, many practices recovered late-incentivised indicators when they were informed only and before financial incentives resumed. Clustering revealed four practice profiles: fast recoverers, fast for incentivised, fast for informed, and slow recoverers. Smaller practices recovered more quickly and rural practices had milder declines.

**Conclusion:**

Financial incentives accelerated quality-of-care recovery after a system shock, particularly when tailored to indicator types and practice contexts, and complemented by timely information provision. Policymakers could use these findings to guide recovery strategies and strengthen resilience in primary care.

## How this fits in

Financial incentives (pay for performance) and information feedback are widely used to improve primary care quality, but their separate effects — especially after major system disruptions such as the COVID-19 pandemic — are unclear. In this study of 287 primary care practices in Catalonia (which served 5 250 531 people), early reinstatement of financial incentives led to faster quality-of-care recovery; information alone was linked to gradual gains in some settings. Indicator type and practice context also influenced the patterns of recovery. Overall, the results support combining and tailoring financial and non-financial strategies to context in order to ensure primary care systems are more resilient.

## Introduction

Healthcare systems must enhance care quality while ensuring financial sustainability; this requires adaptable strategies during shocks such as the COVID-19 pandemic, which strained capacity and disrupted non-pandemic services.^1,2^ Primary care, which is central to triage and chronic disease management, was particularly affected.^3–6^

Among quality-improvement models, pay-for-performance (P4P) schemes link provider reimbursement to measurable outcomes,^7–9^ aiming to align professional incentives with organisational and societal goals.^10–12^ Evidence of effectiveness is mixed:^13–22^ some studies report short-term improvements,^23,24^ particularly for targeted indicators,^12–15,25–31^ while concurrent interventions (for example, public reporting, updated guidelines) may obscure incentive effects.^11,14,15,22^ Slow progress in P4P design and evaluation underscores the need for further evidence, including how financial incentives interact with non-financial strategies.^32,33^

In Catalonia, Spain, the public primary care provider Institut Català de la Salut (ICS) serves ~80% of a population of >8 million people. Since 2006, ICS has combined online feedback about quality indicators with a P4P scheme (Management by Objectives, Direcció per Objectius [DPO]).^34–36^ In 2020, COVID-19 caused declines in chronic disease management;^37,38^ this promped the suspension of the P4P programme, although quality monitoring continued. Financial incentives were reinstated in phases: 15 priority indicators were reintroduced in 2021, with full re-implementation occurring by 2023. This created a natural experiment to analyse:

- the differential effects of performance feedback versus financial incentives on the recovery of primary care quality; and
- how these effects vary by practice characteristics and patient demographics.

This study aimed to analyse how financial and non-financial mechanisms influence recovery from an acute system shock; it addresses a gap in prior research, as that has largely focused on quality improvement under routine conditions. Such findings could inform more targeted and resilient quality improvement strategies for future crises.

## Method

### Study design

This retrospective longitudinal study used data from 37 quality-of-care indicators across 287 primary care practices managed by ICS (from 2019 until 2024). Some indicators were re-incentivised in 2021 (early incentivised), but most were re-incentivised in 2023 (late incentivised), having only been reported to professionals until then. Recovery was assessed via:

- year-on-year percentage change (mean percentage change and coefficient of variation [CV]);
- intra-annual percentage change (January–December); and
- time to recovery (the months from January 2021 until results returned to the pre-pandemic range).

The relationship between incentive status and recovery time was analysed using a mixed-effects Cox proportional hazards model. Unsupervised clustering identified recovery patterns among practices. All 287 practices managed by ICS with complete indicator reporting were included; as the study relied on routinely collected practice-level administrative data, no additional sampling or recruitment pathway of individual professionals or patients was required.

### Data sources and outcome calculations

Monthly indicator data from the Estàndard de Qualitat Assistencial (EQA, Healthcare Quality Standard) framework^34^ were obtained at the practice level from the Sistema d'Informació del Serveis d'Atenció Primària in Catalan (SISAP, Primary Care Services Information System) databases.^39^ The study covered January 2019–December 2024, with 2019 as the pre-pandemic reference. SISAP provided indicator features (for example, incentive status, type) and practice characteristics (for example, socioeconomic deprivation index classification using the MEDEA index,^40^ rurality, demographics, workforce size). As these routinely monitored indicators are validated and curated for clinical and management purposes, no missing data were identified for the selected indicators or practices during the study period.

Time to recovery was calculated projecting 2019 monthly results, except for one indicator (EQA0403) related to home care (2018 values [Supplementary Table S1 and Supplementary Figures S1–S2]). Recovery was the number of months from January 2021 until the mean average practice result (with 95% confidence interval [CI]) re-entered the 2019 range and remained within that range for 6 consecutive months. Indicators unaffected by the pandemic showed negative recovery times; those that had not recovered by December 2024 were assigned 60 months for graphical/clustering use, but excluded from other analyses. For the practice-level analysis, 2019 projections used each practice’s monthly values, with 95% CIs from the standard deviation across all practices.

### Indicator selection

Indicators were included if they:

- had high EQA evidence;^34^
- were financially incentivised for ≥5 years before the pandemic;
- focused on adults (aged >14 years); and
- were re-incentivised between 2021 and 2024.

The 37 selected indicators (Supplementary Table S1) spanned control, diagnosis, follow-up, screening, treatment, and vaccination (Supplementary Table S2). Of these, 13 (35%) were re-incentivised in 2021 (early incentivised: control, *n* = 7; screening, *n* = 3; treatment*, n* = 3). The remaining two of the 15 priority indicators were excluded based on the selection criteria. Although 2021 P4P funds were, ultimately, distributed universally as a pandemic reward, professionals were exposed to the P4P stimulus and unaware of the distribution change until the last quarter of 2021. The remaining 24 indicators (65%) were informed, but unincentivised until 2023 (late incentivised [Supplementary Table S1]: diagnosis, *n* = 7; treatment*, n* = 7; screening*, n* = 5; control*, n* = 2; follow-up, *n* = 2; vaccination, *n* = 1).

### Statistical analysis

A mixed-effects Cox proportional hazards model estimated the effect of incentives on recovery likelihood, accounting for covariates and data hierarchy. Covariates were selected via bivariate analyses (Supplementary Table S3). The following model was used:


$$h_{ij}(t)=h_{0k}(t)\;exp(\beta_{1}IncentiveScheme_{ij}\;+\;\beta_{2}\%ResultDrop_{ij}\;+b_{0j})$$

In the model:

-
$h_{ij}\left(t\right)$ is the recovery hazard at time t for indicator i in practice j ;
-
$h_{0k}\left(t\right)$ is the baseline hazard by indicator type k ;
-
$IncentiveScheme_{ij}$ is the incentive exposure;
-
$\%Resultdrop_{ij}$ is the post-COVID-19 drop; and
-
$b_{0j}∼N\left(0,\sigma^{2}\right)$ is a practice-level random intercept.

The model was stratified by indicator type and included a random effect for practices. Schoenfeld residuals were inspected to assess proportional hazards (Supplementary Figure S3).

*K*-means clustering (R Stats Package) of practice-level recovery times was applied separately for early- and late-incentivised indicators. The optimal number of clusters (*K* = 2) was chosen using within-cluster sum of squares and silhouette width, classifying practices into four recovery profiles:

- fast recoverers;
- fast for incentivised;
- fast for informed; and
- slow recoverers.

Profiles were characterised by contextual and organisational variables.

Descriptive statistics summarised trends. Normality guided parametric (*t*-tests, analysis of variance) or non-parametric (Wilcoxon, Kruskal–Wallis) tests for group comparisons. Spearman’s rank correlation assessed continuous associations, and χ^2^ or Fisher’s exact tests were used for categorical comparisons. False discovery rate correction addressed multiple testing, with significance set at 0.05. Analyses and visualisations were performed in R (version 4.3.1).^41^

### Patient and public involvement

This study used pre-existing administrative aggregate data from pre-established interventions, thereby limiting opportunities for meaningful public engagement. Given the retrospective nature and focus on existing interventions, it was decided not to integrate patient and public involvement in the study.

## Results

### The impact of early incentivisation on initial indicator responses

Quality-of-care indicator data from 287 practices in Catalonia were analysed (Supplementary Table S4). Of these practices, 100 (34.8%) were rural and 187 (65.2%) were urban; 56 (19.5%) practices were in areas of high deprivation. The average population per practice was 18 295 (21 318 in urban practices versus 12 641 in rural practices, *P*<0.001, Supplementary Table S4). The mean patient age was 49 years (48.5 years in urban areas versus 50 years in rural areas, *P*<0.001), 50.6% of patients were females (51.3% in urban areas versus 49.3% in rural areas, *P*<0.001), and 18.4% were immigrants (19.8% in urban areas versus 15.9% in rural areas, *P*<0.001, Supplementary Table S4). Practices averaged 26.6 professionals (29.8 in urban areas and 20.7 in rural areas, *P*<0.001, Supplementary Table S4).

**Figure 1.**
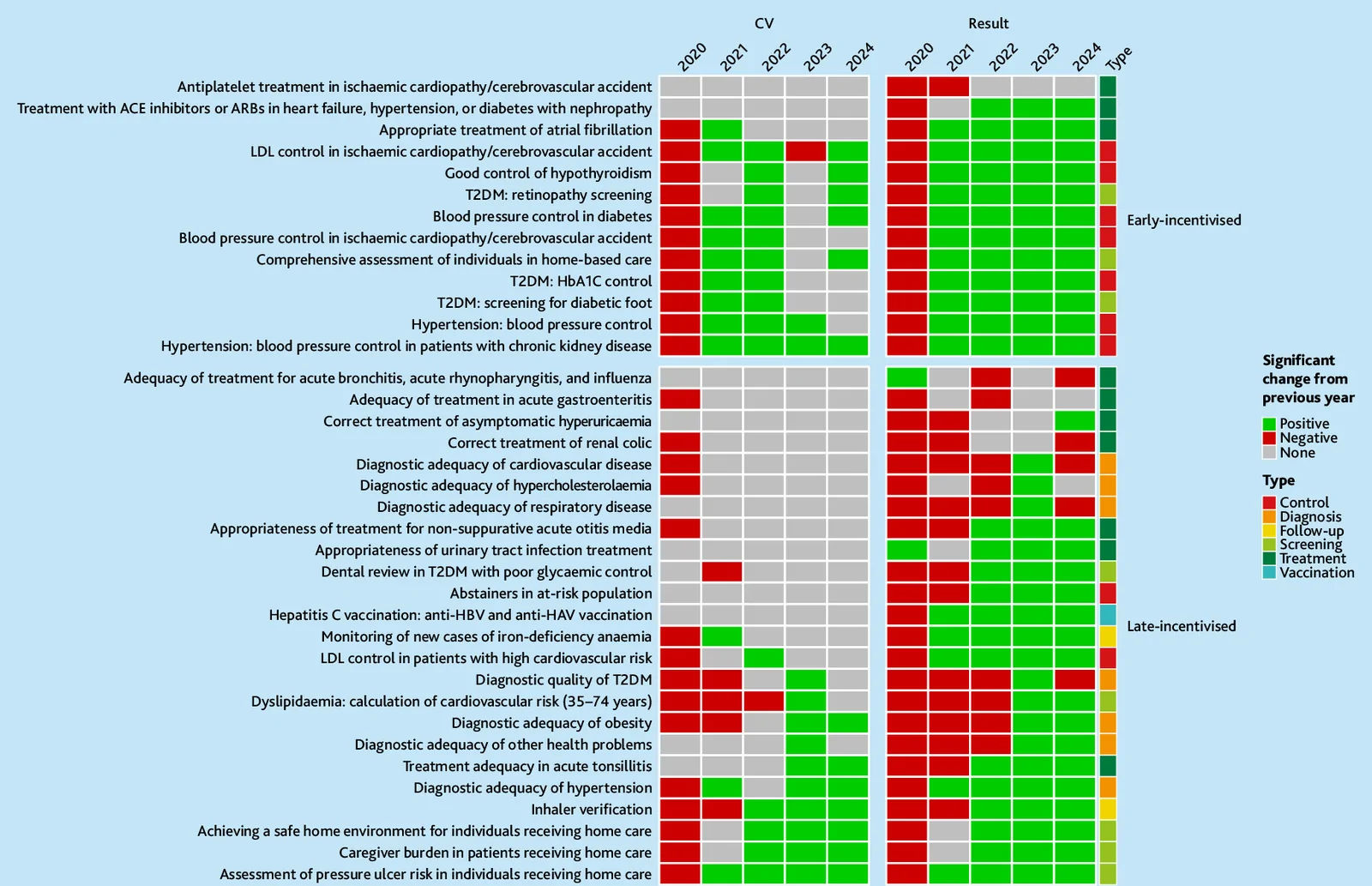
Year-on-year percentage change for all assessed indicators in terms of indicator CV and results across practices. ACE = angiotensin-converting enzyme. ARB = angiotensin receptor blocker. CV = coefficient of variation. HAV = hepatitis A virus. HbA1c = glycated haemoglobin. HBV = hepatitis B virus. LDL = low-density lipoprotein. T2DM = type 2 diabetes mellitus.

In 2020, 35 (95%) of 37 indicators showed a statistically significant drop in results and/or increased variability (CV) when compared with 2019 levels (average 15.3% drop and 115.6% CV increase; Figure 1, Supplementary Figure S4); only two treatment indicators did not. Early-incentivised indicators started recovering sooner than late-incentivised indicators: by December 2021, 11 (85%) of 13 showed improvements, which was defined as having a statistically significant increase in indicator value and/or decrease in variability compared with the previous year (Figure 1). This rose to 12 (92%) out of 13 in 2022, which sustained gains through to 2024 (Figure 1, Supplementary Figure S4). In contrast, late-incentivised indicators improved later: only five (21%) of 24 improved by 2021, increasing to 13 (54%) in 2022, when the inicators were still only informed. Once reintroduced to P4P in 2023, 20 (83%) of 24 late-incentivised indicators improved. (Figure 1, Supplementary Figure S4). Intra-annual changes mirrored this trend: 12 (92%) of 13 early-incentivised indicators improved from January to December 2021, and all improved in 2022. For late-incentivised indicators, intra-annual improvements rose from eight (33%) out of 24 in 2021 to 22 (92%) out of 24 in 2023 (Supplementary Figures S5 and S6).

### Recovery times among early- and late-incentivised indicators

**Figure 2.**
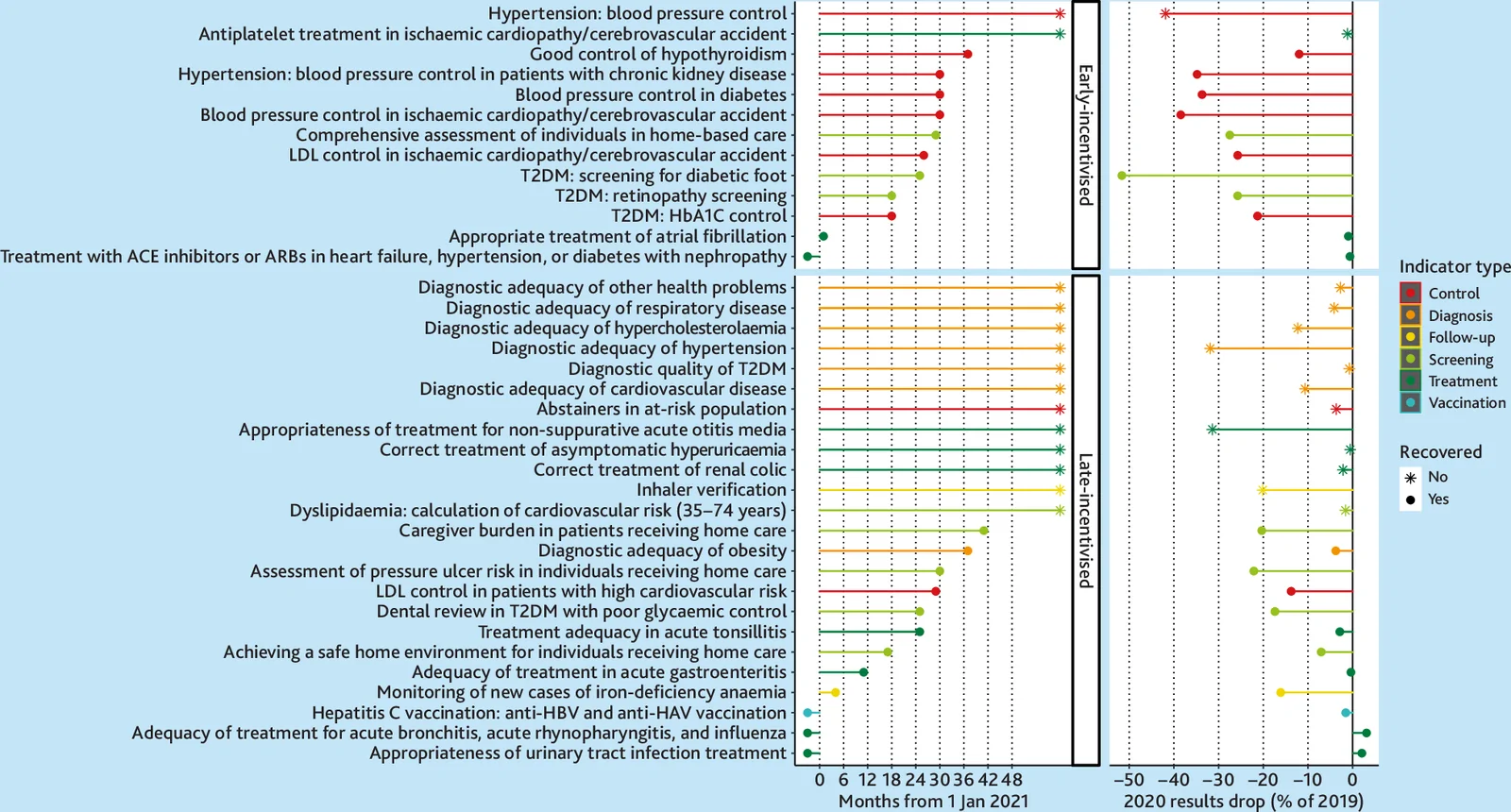
Time to recovery and the drop in results in 2020, all indicators.^a^ ^a^Lollipop plot representing the average recovery times (in months since January 2021) for all indicators (left panel, faceted by financial incentive scheme) and the average percentage drop in results during the COVID-19 pandemic (right panel). HAV = hepatitis A virus. HbA1c = glycated haemoglobin. HBV = hepatitis B virus. LDL = low-density lipoprotein. T2DM = type 2 diabetes mellitus.

Differences in indicators’ average recovery times between treatment arms were assessed Figure 2, and Supplementary Figures S1 and S2). By December 2024, 11 (85%) out of 13 early-incentivised indicators had returned to 2019 levels, compared with 12 (50%) of 24 late-incentivised indicators (Figure 2). Among recovered indicators, average recovery times were not significantly different (21.9 months for early-incentivised indicators versus 17.5 months for late-incentivised indicators, *P* = 0.45); however, treatment indicators recovered significantly faster than control indicators (4.7 months versus 28.6 months respectively, *P*<0.05, Figure 2).

Early-incentivised indicators, although showing higher recovery rates than the late-incentivised ones, also experienced steeper 2020 declines (average percentage drop: −24.3% in early- versus −9.2% in late-incentivised indicators, *P*<0.05), suggesting that the impact the COVID-19 pandemic had on those was greater (Figure 2). The severity of the drop also varied by indicator type: control, screening, and follow-up indicators saw the largest average declines (average 25%, 21.7%, and 18.1% drops in 2019 results, respectively), while diagnosis, treatment, and vaccination indicators were less affected (average 9.4%, 3.5%, and 1.5% drops respectively; *P*<0.05 for treatment versus control or screening [Figure 2]).

**Figure 3.**
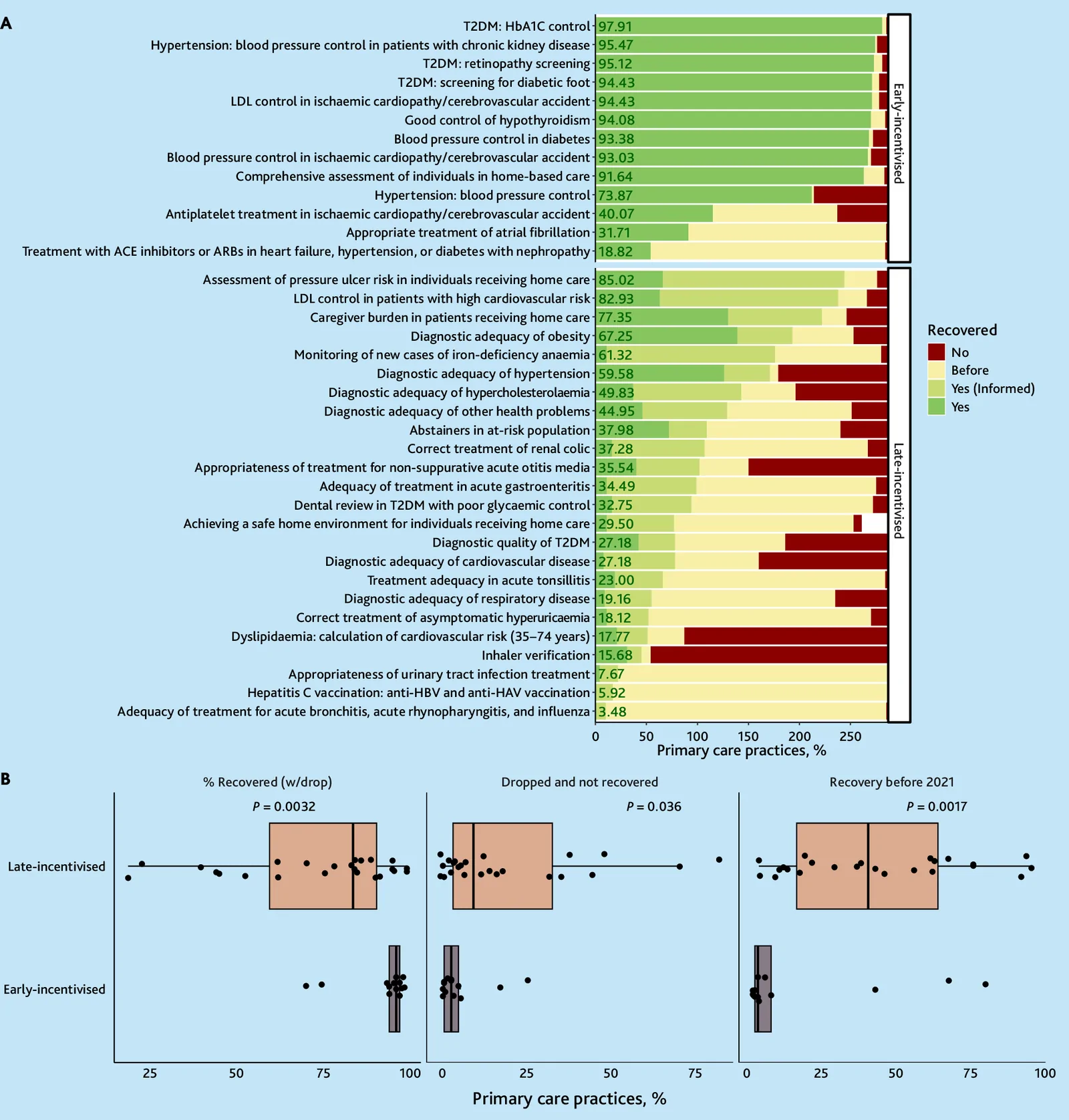
Percentage of practices that recovered/did not recover per indicator: A) Number of practices per indicator and recovery profile.^a^ B) Boxplots comparing the differences in the percentage of practices with different recovery profiles.^b^ ^a^Numbers represent the percentage of recovered practices per indicator. ^b^ *P*-values represent the result of Wilcoxon–Mann–Whitney tests. ACE = angiotensin-converting enzyme. ARB = angiotensin receptor blocker. HAV = hepatitis A virus. HbA1c = glycated haemoglobin. HBV = hepatitis B virus. LDL = low-density lipoprotein. T2DM = type 2 diabetes mellitus.

Using the recovery times across practices (Supplementary Figure S7), for each indicator, the study identified practices that recovered their results by December 2024, those that did not, and those that had recovered before January 2021 (Figure 3A-B). Early-incentivised indicators featured higher numbers of practices dropping results during the pandemic (a lower percentage of practices recovered before 2021 versus findings relating to late-incentivised indicators, *P*<0.01, Figure 3B). However, a higher percentage of the practices that dropped early-incentivised indicator results recovered them before December 2024 (*P*<0.01 when compared with late-incentivised indicators, Figure 3B). Although late-incentivised indicators featured more practices that were unable to recover results in the study period (*P*<0.05 when compared with early-incentivised indicators, Figure 3B), a higher percentage of the practices that recovered the results of late-incentivised indicators did so before 2023, only when they were informed (*P*<0.001, Supplementary Figure S8a).

Further stratification by indicator type revealed differences in recovery patterns (Supplementary Figure S8b): although most practices had already showed recovered results for the treatment and vaccination indicators during 2020, this was not the case for the rest of the indicators. Among control and screening indicators, almost all the practices that were incentivised early were able to recover dropped results, but those that were incentivised late had higher rates of practices that did not recover. Around a third of practices did not manage to recover diagnostic indicator results, all of which were incentivised in 2023.

To assess the impact of early financial incentivisation on the recovery of quality-of-care indicators, a mixed-effects Cox proportional hazards model was fit using recovery time data (per indicator and practice) from January 2021 until December 2022. During this period, late-incentivised indicators remained informed, allowing a direct comparison between incentivised and informed indicators. Initial bivariate analyses (Supplementary Table 3) showed a significantly greater drop in results among incentivised indicators — which were also more often control indicators — with no diagnostic or follow-up indicators represented. The Cox model was stratified by indicator type, included incentive schemes and 2020 result drops as covariates, as well as a random effect for primary care practice to account for clustering and unmeasured heterogeneity. Indicators that remained incentivised during the 2021–2022 period had a significantly higher likelihood of recovery (hazard ratio [HR] = 2.06; 95% CI = 1.88 to 2.25), indicating a 106% greater probability of recovery at any time point than informed-only indicators, after adjusting for baseline drop and clustering. Greater 2020 result drops were associated with slower recovery (HR = 0.988; 95% CI = 0.986 to 0.990).

### Recovery patterns among practices

**Figure 4.**
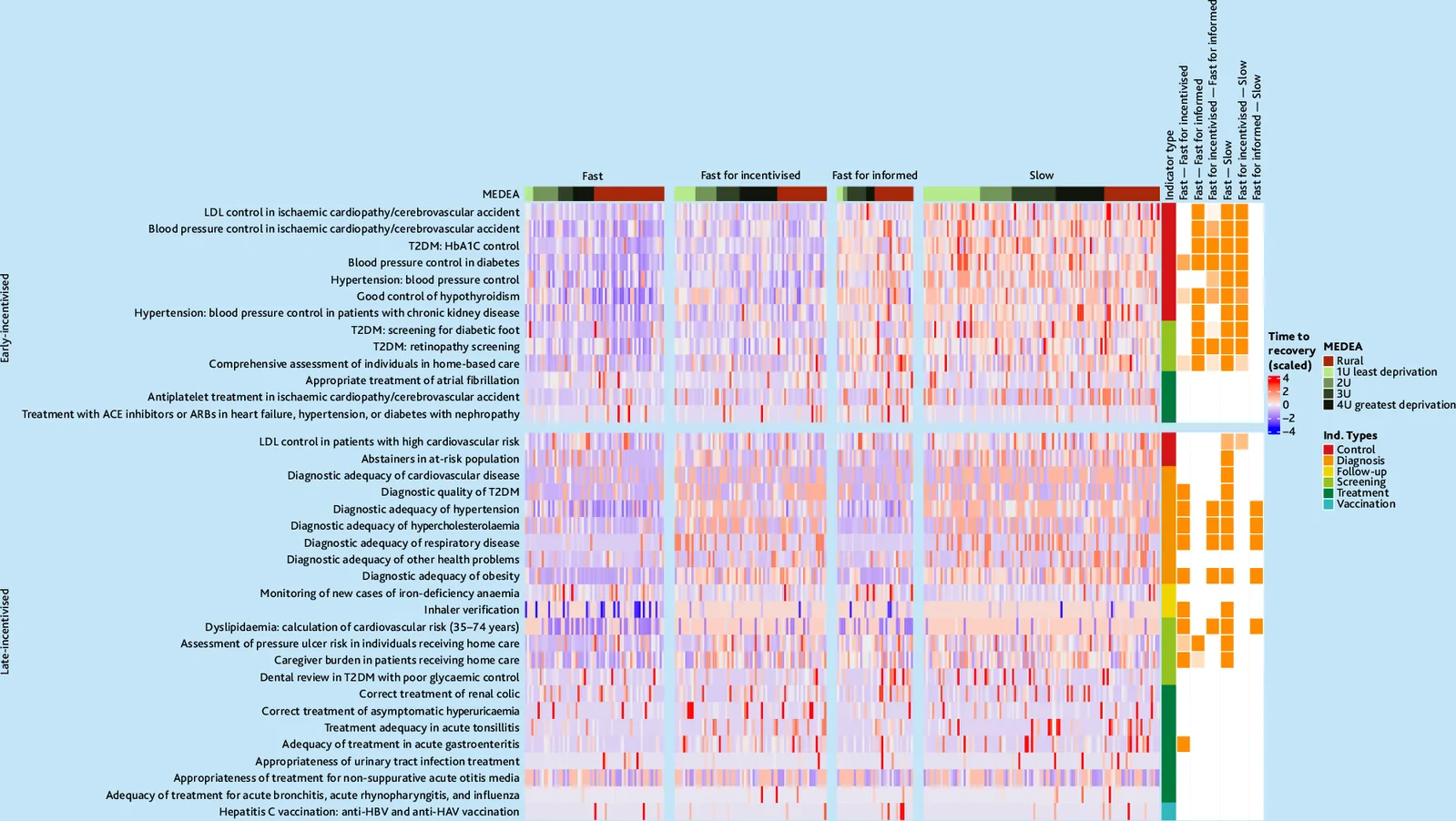
Classification of practices based on recovery patterns found with unsupervised clustering.^a^ ^a^Top annotations indicate rural/urban status and the MEDEA socioeconomic deprivation index (ranked 1U to 4U). Annotations on the right-hand side of the diagram represent indicator types and statistically significant differences in indicator recovery times among clusters: columns represent paired comparisons, and the degree of orange is proportional to a *P*-value significance of <0.05 (Dunn’s post-hoc test) (a darker shade of orange represents a higher level of statistical significance). ACE = angiotensin-converting enzyme. ARB = angiotensin receptor blocker. HAV = hepatitis A virus. HbA1c = glycated haemoglobin. HBV = hepatitis B virus. LDL = low-density lipoprotein. T2DM = type 2 diabetes mellitus.

To identify recovery patterns across practices, unsupervised *K*-means clustering was applied to 2021–2024 recovery time data by practice and indicator. Based on results across incentivised and informed indicators, four practice clusters were defined:

- fast recoverers (short recovery times for both types of indicators);
- fast for incentivised;
- fast for informed; and
- slow recoverers (with the longest overall recovery times) (Figure 4).

Fast and fast for incentivised recoverer practices showed quicker recovery for 10 (77%) out of 13 early-incentivised indicators (*P*<0.05 versus other clusters, Figure 4). Fast recoverers also recovered 13 (54%) of 24 of the late-incentivised indicators (12 indicators versus slow and 10 versus fast for incentivised practices*, P*<0.05), spanning six diagnostic, three screening, two control, one follow-up, and one treatment indicator (Figure 4). Fast for informed recoverers showed better recovery only in five (21%) of 24 indicators (four diagnostic and one screening). No consistent differences in recovery times were observed between clusters for the 10 treatment indicators, where many practices had already recovered by 2021 (Figure 3A, Figure 4).

Cluster characterisation (Table 1, Supplementary Figure S9) revealed enrichment of rural practices among fast and fast for informed clusters (50% each versus 32% and 23% of fast for incentivised and slow practices respectively [Table 1, Supplementary Figure S9A]). Fast recoverers had smaller assigned populations (average 16 230 versus 20 348 in slow practices), fewer professionals (24.8% versus 28.6% in slow practices), and lower 2020 drops (11% versus 14.7–16% among the rest, Table 1). These lower drops were mainly attributable to fast-recovering rural practices, with no statistically significant drop differences among urban practices in areas of least deprivation (Supplementary Figure S9B).

**Table 1.** Summary of the associations between recovery profiles and the sociodemographic and organisational characteristics of practices^a^

	Fast, *n = 66*	Fast for incentivised, *n =* 72	Fast for informed, *n =* 36	Slow, *n =* 112	Overall *P*-value
**MEDEA socioeconomic deprivation index^b^**					.
**1U, *n* (%)**	4 (6.06)	10 (13.9)	3 (8.33)	27 (24.1)	
**2U, *n* (%)**	12 (18.2)	10 (13.9)	2 (5.56)	15 (13.4)	
**3U*, n (%)***	7 (10.6)	11 (15.3)	9 (25.0)	21 (18.8)	
**4U, *n* (%)**	10 (15.2)	18 (25.0)	4 (11.1)	23 (20.5)	
**Rural, *n* (%)**	33 (50.0)	23 (31.9)	18 (50.0)	26 (23.2)	
***N* patients, average (sd)**	16 230 (8163)	17 838 (7785)	16 840 (8731)	20 348 (7755)	**0.005**
**Age of patients, average (sd)**	49.5 (1.81)	48.8 (1.73)	48.9 (2.24)	48.9 (1.91)	0.177
**% female patients, average (sd)**	50.2 (1.67)	50.7 (1.58)	50.2 (2.26)	50.9 (2.53)	0.139
**% immigrant patients, average (sd)**	16.7 (7.24)	18.2 (8.88)	20.3 (8.56)	19.1 (9.76)	*0.202*
***N* professionals, average (sd)**	24.8 (10.9)	26.3 (9.66)	24.8 (10.60)	28.6 (9.40)	**0.046**
***N* GPs, average (sd)**	12.3 (5.46)	13.1 (4.97)	12.4 (5.40)	14.4 (4.77)	**0.035**
***N* nurses, average (sd)**	12.5 (5.53)	13.2 (4.74)	12.4 (5.20)	14.3 (4.68)	0.063
**Average percentage drop from 2019 results**	–11.02 (4.70)	–14.83 (4.24)	–14.74 (3.76)	–16.04 (3.67)	**<0.001**

^a^n = number of observations; sd = standard deviation. ^b^MEDEA (Mortality in small areas of Spain and socioeconomic and environmental inequalities) classification stratifies urban practices according to the socioeconomic deprivation of the area they serve, ranked from 1U (least deprivation) to 4U (most deprivation).

## Discussion

### Summary

This study adds to evidence on primary care improvement by evaluating how financial incentives and information provision aided quality-of-care recovery after the system shock of COVID-19, which diverted focus towards pandemic control and reduced routine care.^3,37^ Thirty-seven indicators across 287 practices were analysed; 13 of these indicators were financially incentivised in 2021 and 24 were re-incentivised in 2023, enabling comparison between financial incentives and information-only strategies. Early reinstatement of incentives accelerated care-quality recovery, and doubled the likelihood of recovery being achieved in 2021–2022. Information alone also supported gradual improvements, with just over half of late-incentivised indicators improving before incentives resumed. Indicator type and practice context influenced recovery, highlighting the value of combining and tailoring strategies for resilience.

### Strengths and limitations

This study focused on a setting and context that has been rarely examined in prior research. It involved a large sample, monthly data spanning >5 years, and the natural experiment created by phased incentive reintroduction. The broad indicator set enabled exploration of differential responses by type and practice profile, and clustering offered novel insight into recovery patterns. Limitations included the absence of a contemporaneous control group due to the system-wide rollout. In addition, because indicators were reintroduced in phases, those reinstated earlier were likely prioritised by policymakers on the basis of perceived clinical importance or feasibility.

Uneven indicator distribution (for example, diagnostic, follow-up, and vaccination indicators only featuring in the late-incentivised group) limited full comparisons. The Cox model was stratified by indicator type, allowing the baseline hazard to vary across strata, and was adjusted for relevant covariates. The effect of incentives (expressed as hazard ratios) could only be estimated for indicators observed in both comparison groups. This restriction, together with the proportional hazards assumption, should be considered when interpreting the results.

Although the findings reflect the structure and incentive framework of Catalonia’s public primary care system, their generalisability to other health systems may depend on local organisational contexts, incentive structures, funding models, and recovery strategies. Finally, these findings are framed in a post-pandemic recovery phase, when quality-improvement dynamics and provider behaviour may differ from that which exists in routine periods; it also remains uncertain whether observed improvements can be sustained once incentives are withdrawn.

### Comparison with existing literature

The finding that financially incentivised indicators recovered more quickly, with double the likelihood of recovery during 2021–2022, is consistent with prior research showing that financial incentives can prompt faster behaviour change, particularly with low baseline performance.^14,23,24,42^ More than half of late-incentivised indicators improved during the information-only period, which supports earlier evidence on the sustained benefits of non-financial interventions.^23,24,35^

Unlike earlier studies, which often examined narrower indicator sets,^14,15,43,44^ this analysis covered a broader range, enabling nuanced assessment of recovery patterns by indicator type and practice profile. Consistent with prior evidence that prescribing activity was more amenable to remote delivery during service disruption, we observed relatively faster recovery among treatment indicators, whereas diagnostic indicators recovered more slowly,^37^

Our findings also align with reports of greater resilience among rural and smaller practices, which have been attributed to lower service disruption in more dispersed populations. In contrast, the slower recovery observed in urban practices in our study suggests that these settings may require more targeted or sustained support during system-wide shocks. By examining a broader and more heterogeneous indicator set than earlier studies, our analysis extends existing evidence on differential recovery across service types and practice contexts.^37,45^

### Implications for research and practice

For policymakers, these findings suggest that targeted financial incentives for a reduced set of indicators may support recovery.^46,47^ Indicators should be selected based on clinical relevance, low baseline performance,^14,17^ and contextual barriers. As improvement is context dependent, multifaceted approaches combining financial and non-financial measures may yield better long-term outcomes. Informed indicators have also underscored the value of transparency and clinician engagement, even without direct rewards. Sustained improvement requires regular monitoring, flexible incentive designs, and alignment with meaningful clinical goals and local realities.

Overall, this study examined short- to medium-term effects of financial and informational interventions in a recovery setting. Whether improvements persist without incentives remains unclear, and future research should explore long-term effects and broader generalisability. Although the findings presented here align with evidence supporting the effectiveness of financial incentives,^12,42,48,49^ debates persist about incentives’ overall value and unintended consequences.^14,15^ Continued evaluation across varied contexts is crucial to guide equitable, effective quality-improvement policies.

## Data

The data underlying this study consist of de-identified patient-level healthcare records and are subject to strict governance and data protection regulations. The authors do not have permission to share these data publicly. Access may be granted to qualified researchers subject to approval by the data provider and relevant regulatory bodies. Researchers wishing to access the data should apply directly to ICS, https://ics.gencat.cat/ca/inici, subject to appropriate data use agreements and ethical approval.

## References

[1] Sachs JD, Karim SSA, Aknin L (2022). The Lancet Commission on lessons for the future from the COVID-19 pandemic. Lancet.

[2] Sagan A, Webb E, McKee M (2021). Health systems resilience during COVID-19: lessons for building back better.

[3] Haldane V, De Foo C, Abdalla SM (2021). Health systems resilience in managing the COVID-19 pandemic: lessons from 28 countries. Nat Med.

[4] Oosterhoff M, Kouwenberg LHJA, Rotteveel AH (2023). Estimating the health impact of delayed elective care during the COVID-19 pandemic in the Netherlands. Soc Sci Med.

[5] Whaley CM, Pera MF, Cantor J (2020). Changes in health services use among commercially insured US populations during the COVID-19 pandemic. JAMA Netw Open.

[6] Frey A, Tilstra AM, Verhagen MD (2024). Inequalities in healthcare use during the COVID-19 pandemic. Nat Commun.

[7] Organisation for Economic Co-operation and Development (2010). Value for money in health spending. https://www.oecd.org/en/publications/value-for-money-in-health-spending_9789264088818-en.html.

[8] Panteli D, Quentin W, Busse R, Busse R, Klazinga N, Panteli D (2019). Improving healthcare quality in Europe: characteristics, effectiveness and implementation of different strategies.

[9] Quentin W, Partanen V-M, Brownwood I, Busse R, Klazinga N (2019). Improving healthcare quality in Europe: characteristics, effectiveness and implementation of different strategies.

[10] Roland M, Dudley RA (2015). How financial and reputational incentives can be used to improve medical care. Health Serv Res.

[11] Roland M, Guthrie B (2016). Quality and Outcomes Framework: what have we learnt?. BMJ.

[12] Doran T, Kontopantelis E, Valderas JM (2011). Effect of financial incentives on incentivised and non-incentivised clinical activities: longitudinal analysis of data from the UK Quality and Outcomes Framework. BMJ.

[13] Wagenschieber E, Blunck D (2024). Impact of reimbursement systems on patient care: a systematic review of systematic reviews. Health Econ Rev.

[14] Mendelson A, Kondo K, Damberg C (2017). The effects of pay-for-performance programs on health, health care use, and processes of care: a systematic review. Ann Intern Med.

[15] Eijkenaar F, Emmert M, Scheppach M, Schöffski O (2013). Effects of pay for performance in health care: a systematic review of systematic reviews. Health Policy.

[16] Flodgren G, Eccles MP, Shepperd S (2011). An overview of reviews evaluating the effectiveness of financial incentives in changing healthcare professional behaviours and patient outcomes. Cochrane Database Syst Rev.

[17] Van Herck P, De Smedt D, Annemans L (2010). Systematic review: effects, design choices, and context of pay-for-performance in health care. BMC Health Serv Res.

[18] Scott A, Liu M, Yong J (2018). Financial incentives to encourage value-based health care. Med Care Res Rev.

[19] Witter S, Fretheim A, Kessy FL, Lindahl AK (2012). Paying for performance to improve the delivery of health interventions in low- and middle-income countries. Cochrane Database Syst Rev.

[20] Steel N, Willems S (2010). Research learning from the UK Quality and Outcomes Framework: a review of existing research. Qual Prim Care.

[21] Gillam SJ, Siriwardena AN, Steel N (2012). Pay-for-performance in the United Kingdom: impact of the Quality and Outcomes Framework: a systematic review. Ann Fam Med.

[22] Bujalance-Zafra MJ, Domínguez-Santaella M, Baca-Osorio A (2017). [Analysis of an intervention to improve health outcomes in acute exacerbations of COPD in primary care]. Aten Primaria.

[23] Chauhan BF, Jeyaraman MM, Mann AS (2017). Behavior change interventions and policies influencing primary healthcare professionals’ practice: an overview of reviews. Implement Sci.

[24] Allen T, Whittaker W, Kontopantelis E, Sutton M (2018). Influence of financial and reputational incentives on primary care performance: a longitudinal study. Br J Gen Pract.

[25] Heider A-K, Mang H (2020). Effects of monetary incentives in physician groups: a systematic review of reviews. Appl Health Econ Health Policy.

[26] Kontopantelis E, Springate D, Reeves D (2014). Withdrawing performance indicators: retrospective analysis of general practice performance under UK Quality and Outcomes Framework. BMJ.

[27] Gené-Badia J, Escaramis-Babiano G, Sans-Corrales M (2007). Impact of economic incentives on quality of professional life and on end-user satisfaction in primary care. Health Policy.

[28] Minchin M, Roland M, Richardson J (2018). Quality of care in the United Kingdom after removal of financial incentives. N Engl J Med.

[29] Petersen LA, Simpson K, Pietz K (2013). Effects of individual physician-level and practice-level financial incentives on hypertension care: a randomized trial. JAMA.

[30] Campbell SM, Reeves D, Kontopantelis E (2009). Effects of pay for performance on the quality of primary care in England. N Engl J Med.

[31] Ogundeji YK, Bland JM, Sheldon TA (2016). The effectiveness of payment for performance in health care: a meta-analysis and exploration of variation in outcomes. Health Policy.

[32] Zaresani A, Scott A (2021). Is the evidence on the effectiveness of pay for performance schemes in healthcare changing? Evidence from a meta-regression analysis. BMC Health Serv Res.

[33] Ogundeji YK, Sheldon TA, Maynard A (2018). A reporting framework for describing and a typology for categorizing and analyzing the designs of health care pay for performance schemes. BMC Health Serv Res.

[34] Coma E, Ferran M, Méndez L (2013). Creation of a synthetic indicator of quality of care as a clinical management standard in primary care. Springerplus.

[35] Esteban-Fabró R, Coma E, Hermosilla E (2024). Information provision and financial incentives in Catalonia’s public primary care (2010–2019): an interrupted time series analysis. Lancet Reg Health Eur.

[36] Coma E, Medina M, Méndez L (2019). Effectiveness of electronic point-of-care reminders versus monthly feedback to improve adherence to 10 clinical recommendations in primary care: a cluster randomized clinical trial. BMC Med Inform Decis Mak.

[37] Coma E, Mora N, Méndez L (2020). Primary care in the time of COVID-19: monitoring the effect of the pandemic and the lockdown measures on 34 quality of care indicators calculated for 288 primary care practices covering about 6 million people in Catalonia. BMC Fam Pract.

[38] Coma E, Miró Q, Medina M (2021). Association between the reduction of face-to-face appointments and the control of patients with type 2 diabetes mellitus during the COVID-19 pandemic in Catalonia. Diabetes Res Clin Pract.

[39] Coma E, Méndez Boo LS (2010). 4 años buceando en mares de datos [four years diving into seas of data]. Actualización En Medicina de Familia.

[40] Domínguez-Berjón MF, Borrell C, Cano-Serral G (2008). [Constructing a deprivation index based on census data in large Spanish cities (the MEDEA project)]. Gac Sanit.

[41] R Core Team

[42] Ho L, Mercer SW, Henderson D (2025). Effect of UK Quality and Outcomes Framework pay-for-performance programme on quality of primary care: systematic review with quantitative synthesis. BMJ.

[43] Cattel D, Eijkenaar F (2020). Value-based provider payment initiatives combining global payments with explicit quality incentives: a systematic review. Med Care Res Rev.

[44] Morales DR, Minchin M, Kontopantelis E (2023). Estimated impact from the withdrawal of primary care financial incentives on selected indicators of quality of care in Scotland: controlled interrupted time series analysis. BMJ.

[45] Delgado Viñas C (2023). Los efectos de la pandemia COVID-19 en los espacios rurales: Cantabria (España) como estudio de caso/The COVID-19 pandemic effects in rural areas: Cantabria (Spain) as a case study. Ería.

[46] Lavergne MR, Law MR, Peterson S (2016). A population-based analysis of incentive payments to primary care physicians for the care of patients with complex disease. CMAJ.

[47] Leuchter RK, Sarkisian CA, Trotzky-Sirr R (2023). Choosing wisely interventions to reduce antibiotic overuse in the safety net. Am J Manag Care.

[48] McKay AJ, Gunn LH, Sathish T (2021). Associations between attainment of incentivised primary care indicators and incident diabetic retinopathy in England: a population-based historical cohort study. BMC Med.

[49] Doran T, Fullwood C, Kontopantelis E, Reeves D (2008). Effect of financial incentives on inequalities in the delivery of primary clinical care in England: analysis of clinical activity indicators for the Quality and Outcomes Framework. Lancet.

